# Systemic Levels of Pro-Inflammatory Cytokines and Post-Treatment Modulation in Tuberculous Lymphadenitis

**DOI:** 10.3390/tropicalmed8030150

**Published:** 2023-02-27

**Authors:** Gokul Raj Kathamuthu, Kadar Moideen, Rathinam Sridhar, Dhanaraj Baskaran, Subash Babu

**Affiliations:** 1 National Institutes of Health-NIRT-International Center for Excellence in Research, Chennai 600 031, India; 2 National Institute for Research in Tuberculosis (NIRT), Chennai 600 031, India; 3 Department of Microbiology, Tumor and Cell Biology, Karolinska Institutet, 171 77 Solna, Sweden; 4 Government Stanley Medical Hospital, Chennai 600 001, India; 5 Laboratory of Parasitic Diseases, National Institute of Allergy and Infectious Diseases, National Institutes of Health, Bethesda, MD 20892-0425, USA

**Keywords:** TBL, LTBI, healthy controls, pro-inflammatory cytokines, ELISA

## Abstract

Pro-inflammatory cytokines are potent stimulators of inflammation and immunity and markers of infection severity and bacteriological burden in pulmonary tuberculosis (PTB). Interferons could have both host-protective and detrimental effects on tuberculosis disease. However, their role has not been studied in tuberculous lymphadenitis (TBL). Thus, we evaluated the systemic pro-inflammatory (interleukin (IL)-12, IL-23, interferon (IFN)α, and IFNβ) cytokine levels in TBL, latent tuberculosis (LTBI), and healthy control (HC) individuals. In addition, we also measured the baseline (BL) and post-treatment (PT) systemic levels in TBL individuals. We demonstrate that TBL individuals are characterized by increased pro-inflammatory (IL-12, IL-23, IFNα, IFNβ) cytokines when compared to LTBI and HC individuals. We also show that after anti-tuberculosis treatment (ATT) completion, the systemic levels of pro-inflammatory cytokines were significantly modulated in TBL individuals. A receiver operating characteristic (ROC) analysis revealed IL-23, IFNα, and IFNβ significantly discriminated TBL disease from LTBI and/or HC individuals. Hence, our study demonstrates the altered systemic levels of pro-inflammatory cytokines and their reversal after ATT, suggesting that they are markers of disease pathogenesis/severity and altered immune regulation in TBL disease.

## 1. Introduction

Tuberculosis (TB) remains an important and key public health problem caused by Mycobacterium tuberculosis (Mtb). According to World Health Organization (WHO) estimation, worldwide 10.6 million individuals fell ill with TB in the year 2021 with reported 1.4 million deaths in human immunodeficiency virus (HIV) negative individuals and 1.6 million deaths in HIV-positive individuals [1]. The involvement of extra-pulmonary tuberculosis (EPTB) is about 15–20% in HIV-negative patients and 40–50% in HIV-positive patients, among all types of TB disease [2]. The most typical form of EPTB is tuberculous lymphadenitis (TBL) which chiefly affects the cervical region of the lymph nodes (LNs), followed by skin, bone, abdominal, genitourinary, cerebrospinal, meninges, and joints [3,4]. The EPTB disease proportion differs from pulmonary TB (PTB) among nations and varies based on the associated illnesses and ethnicity [5]. The protective immune-mediated responses in PTB are now well established and require both innate as well as cellular immunity and antigen presentation by dendritic cells, which are essential for host resistance [6,7]. In addition, pro-inflammatory (granulocyte-macrophage colony-stimulating factor (GMCSF), IL-1α, IL-β, tumor necrosis factor (TNF)α, IFNγ) cytokines play an important role in facilitating the host immune protection against TB disease, as shown by animal studies [8,9,10]. They are mostly associated with the expression of immune responses, involvement in macrophage activation, stimulation of apoptosis, and recruitment of other immune cells [7,11].

Interleukin (IL)-12 family cytokines (IL-12, IL-23, IL-27, IL-35) are generally secreted by antigen-presenting cells (APCs) during T cell antigen presentation [12,13,14]. They provide the link between innate and cellular immunity by priming naïve CD4+ T cells [15]. IL-12 effectively induces strong immunological effects upon the phagocytosis of Mtb through macrophages and dendritic cells (DCs), which are essential for the stimulation of T helper (Th) 1 cells [16]. Both IL-12 and IL-1β are produced at lesser levels by DCs after Mtb infection [10]. IL-23 may also encourage the differentiation of Th17 cells in the LNs primarily in conjunction with IL-6, IL-1β, and TGFβ cytokines [10]. Similarly, a previous study indicated the role of IL-23 in providing a protective immune response against mycobacterial infection. Thus, mice lacking IL-12/IL-23 cytokines are more vulnerable to Mtb disease compared to mice lacking IL-12 cytokine due to their reduced ability to induce IFNγ production [17]. IL-23 deficient mice mount a poor immune response against Mtb infection [18]. After Mtb infection, the comparative levels of IL-12 and IL-23 will determine the stability of Th1 and Th17 cells [18]. Earlier studies have reported that IL-12, IL-23, and IL-27 cytokines could act locally as an adjuvant for DNA vaccines, and both IL-12 and IL-23 mediate cellular proliferation, the polarization of Th1 cells, and provide resistance after immunization [19,20,21,22].

Mtb infection stimulates type I interferon (IFN) expression in humans and is also expressed in the myeloid dendritic cells and macrophages of mice [23]. They are activated ubiquitously in innate immune cells and could mediate either defensive or harmful functions against bacterial infection and viral diseases via inflammation or immunosuppression [24,25]. Hence, type I IFNs are useful for the treatment of many diseases, such as hepatitis B/C infections, multiple sclerosis, various malignancies, and adjuvants for active TB. They are powerful inhibitors of IL-12 cytokine production via human monocytes/macrophages, a cytokine requisite for IFNγ production [26]. Recently, the blood transcriptional profile dominated by a type I IFN signature in human active TB disease was shown to correlate with extended disease pathology [24,27,28].

Pro-inflammatory cytokines mediate resistance as well as susceptibility; whether differences in the infection site (lungs versus cervical lymph nodes) could cause an alteration in the inflammatory response is unknown. More importantly, the systemic pro-inflammatory (IL-12, IL-23, IFNα, IFNβ) cytokine levels in TBL disease have not been explored and, thus, we have explored the same in our present study. Our results describe how TBL individuals exhibit elevated pro-inflammatory cytokines which are reversed after anti-tuberculosis (ATT) treatment.

## 2. Materials and Methods

### 2.1. Statement of Ethics

The ethical approval was obtained from the Institutional Ethical Committee (IEC2010007) affiliated with the National Institute for Research in Tuberculosis (NIRT) and written consent was obtained from the study participants. The samples were collected between the years 2013 to 2018.

### 2.2. Research Participants

In this study, a set of 123 individuals with TBL (*n* = 40), LTBI (*n* = 40), and HC (*n* = 43) was included. The demographics are illustrated in Table 1. The TBL group was diagnosed as positive based on bacteriological culture or histology performed on the LN tissue. The samples were collected before the commencement of the treatment. The inclusion study criteria were cervical lymph node TB patients who were positive either by GeneXpert or culture or histology and without a prior history of TB disease. Similarly, the exclusion criteria were patients who were seropositive for the HIV virus and active TB disease (pulmonary TB). The TBL group was treated with 6 months of the standard anti-TB drugs and samples were also redrawn after treatment. The TBL disease cure was defined by the complete resolution of cervical lymph node enlargement. The positivity of LTBI infection was confirmed by the tuberculin skin test (TST), interferon-gamma release assay (IGRA), and the nonappearance of pulmonary signs on the chest radiograph. We considered more than 12 mm in diameter as a positive TST result for the LTBI group to minimize the false positive results owing to environmental mycobacteria exposure. HCs were defined based on the negative results obtained from both TST (induration diameter <5 mm) and QuantiFERON TB-Gold in Tube ELISA (Qiagen, Valencia, CA, USA). The study participants were HIV-negative and not on any steroid treatments.

### 2.3. Enzyme-Linked Immunosorbent Assay (ELISA)

The plasma was separated from the peripheral blood samples and stored at −80 °C after centrifugation for 20 min at 2600 revolutions per minute at 4 °C. The systemic levels of pro-inflammatory (IL-12, IL-23, IFNα, IFNβ) cytokines were measured using the Duoset sandwich ELISA kits (R&D Systems). Briefly, the plate was coated overnight with a capture antibody. After incubation, the plates were washed and blocked in a phosphate buffer saline/bovine serum albumin followed by samples, and standards were added and incubated for 2 h at room temperature. After, the detection antibody was added and incubated for 2 h, followed by streptavidin-horseradish peroxidase and incubated for 20 min. After each step, the samples were aspirated from the plates and washed three times with the wash buffer. Then, the substrate was added and incubated for 20 min in the dark at room temperature. Finally, a stop solution was added and the optical density was measured at 450 nm. The samples below the detection limit were extrapolated using a curve fit and used for analysis. All the samples were run together in a single run without a time gap and in duplicates.

### 2.4. Analysis

We utilized the Graph-Pad PRISM (Version 9, San Diego, CA, USA) software to perform statistical analyses. Geometric means (GM) were deployed to analyze the central tendency. The significant differences between TBL, LTBI, and HC individuals were calculated using the Kruskal-Wallis test with Dunn’s post hoc test for multiple comparisons. The baseline and post-treatment responses among TBL individuals were analyzed using the Wilcoxon signed-rank test. We measured the power of each marker by receiver operator characteristic (ROC, MedCalc Software) to distinguish the TBL from LTBI and HC individuals. A multiple linear regression (non-parametric spearman correlation) analysis for each cytokine was performed between TBL versus LTBI and TBL versus HC individuals.

## 3. Results

### 3.1. TBL Group Has Increased Pro-Inflammatory Cytokine Levels

**Figure 1:**
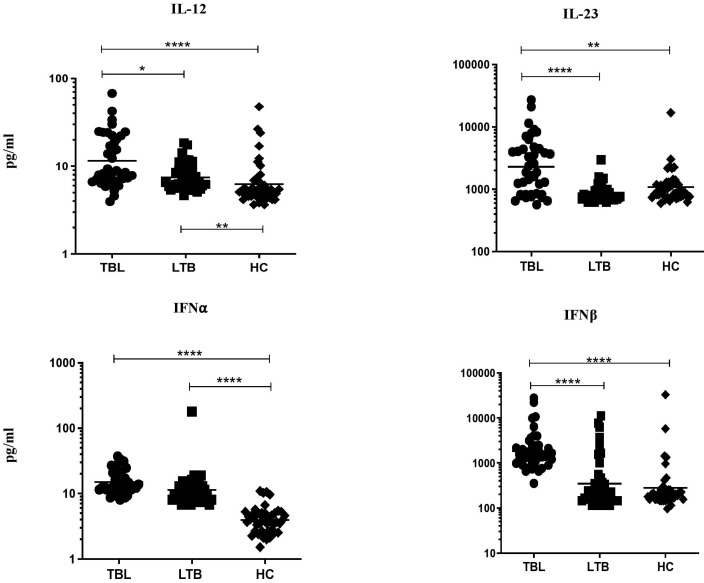
TBL group has increased systemic levels of pro-inflammatory cytokines. We used ELISA to measure the systemic levels of pro-inflammatory (IL-12, IL-23, interferons (IFN)α, and IFNβ) cytokines in TBL (*n* = 40), LTBI (*n* = 40), and HC (*n* = 43) individuals. The figures were illustrated as scatter plots and each individual was shown as a circle or square or diamond, with the bars indicating the geometric mean. We deployed Kruskal-Wallis test with Dunn’s post hoc test for multiple comparisons to calculate the *p*-values. The star represents the significance levels (* *p* < 0.05, ** *p* < 0.01, and **** *p* < 0.0001) between the study groups.

The pro-inflammatory cytokine levels (IL-12, IL-23, IFNα, and IFNβ) were examined among the TBL, LTBI, and HC groups (Figure 1). The systemic levels of IL-12 (geometric mean (GM) of TBL is 11.50 pg/mL versus (vs.) 7.464 pg/mL in LTBI vs. 6.239 pg/mL in HC), IL-23 (GM of TBL is 2297 pg/mL vs. 860.2 pg/mL in LTBI vs. 1085 pg/mL in HC), IFNα (GM of TBL is 15.02 pg/mL vs. 11.35 pg/mL in LTBI vs. 3.927 pg/mL in HC), and IFNβ (GM of TBL is 1841.0 pg/mL vs. pg/mL in 346.9 LTBI vs. 278.2 pg/mL in HC) cytokines were significantly increased in the TBL group compared with the LTBI and HC groups. Hence, our data reveals that increased pro-inflammatory cytokines are the characteristic features of TBL disease.

### 3.2. Anti-Tuberculosis Treatment Effect on Pro-Inflammatory Cytokines

**Figure 2:**
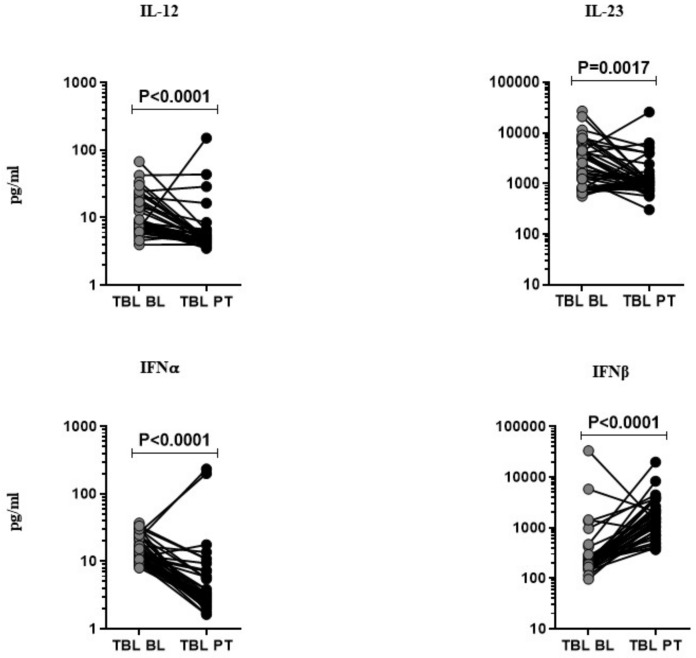
Effect of anti-tuberculosis treatment in TBL disease. The systemic (baseline (BL) and PT) levels of pro-inflammatory (IL-12, IL-23, IFNα, and IFNβ) cytokines were measured by ELISA in TBL (*n* = 40), individuals. We display the figures using a line graph and each line denotes a single individual. Wilcoxon signed-rank tests were deployed to calculate *p*-values.

The pro-inflammatory cytokines (IL-12, IL-23, IFNα, and IFNβ) were measured at pre- and post-treatment time points after the completion of the anti-tuberculosis treatment (ATT) in TBL individuals (Figure 2). Among them, IL-12 (GM of TBL post-treatment is 5.978 pg/mL vs. 11.50 pg/mL in pre-treatment), IL-23 (GM of TBL post-treatment is 1260.0 pg/mL vs. 2297.0 pg/mL in pre-treatment), and IFNα (GM of 5.116 pg/mL in post-treatment vs. 15.02 pg/mL in pre-treatment) cytokine levels were significantly lower in TBL at the post-treatment time point in comparison with the baseline levels. In contrast, the IFNβ cytokine levels were significantly elevated in the TBL post-treatment compared to the baseline. Hence, pro-inflammatory cytokines were modulated upon the successful completion of ATT.

### 3.3. ROC Analysis of Pro-Inflammatory Cytokines in TBL Disease

**Figure 3:**
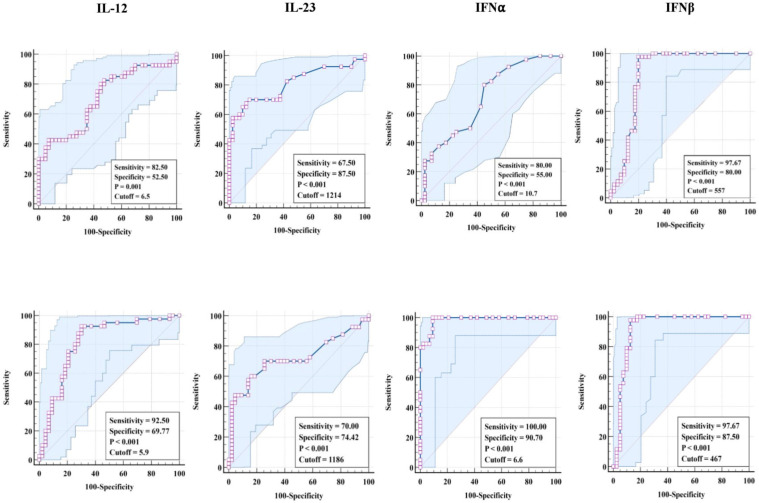
ROC analysis of pro-inflammatory cytokines. The analysis was deployed to calculate the sensitivity, specificity, and cut-off values for (**upper row** TBL (*n* = 40) against LTBI (*n* = 40) and (**lower row**) TBL against HC individuals (*n* = 43).

We explored the discriminatory potential of pro-inflammatory cytokines to discriminate TBL from LTBI and HC groups by ROC analysis. Our results exhibit that pro-inflammatory (IL-12 [sensitivity, 82.5%; specificity, 52.5%; *p* = 0.001; cut-off = 6.5], IL-23 [sensitivity, 67.5%; specificity, 87.5%; *p* < 0.001; cut-off = 1214], IFNα [sensitivity, 80%; specificity, 55%; *p* < 0.001; cut-off = 10.7], and IFNβ [sensitivity, 97.67%; specificity, 80%; *p* < 0.001; cut-off = 557] cytokines exhibit significant sensitivity and specificity in discriminating TBL from LTBI individuals (Figure 3 [upper row]). Similarly, pro-inflammatory (IL-12 [sensitivity, 92.5%; specificity, 69.7%; *p* < 0.001; cut-off = 5.9], IL-23 [sensitivity, 70%; specificity, 74.42%; *p* < 0.001; cut-off = 1186], IFNα [sensitivity, 100%; specificity, 90.70%; *p* < 0.001; cut-off = 6.6], and IFNβ [sensitivity, 97.67%; specificity, 87.5%; *p* < 0.001; cut-off = 467]) cytokines exhibit significant sensitivity and specificity in discriminating TBL from HC individuals (Figure 3 [lower row]). Thus, pro-inflammatory cytokines possess the ability to discriminate TBL from LTBI and HC individuals.

### 3.4. Correlation Analysis of Pro-Inflammatory Cytokines in TBL Disease

**Figure 4:**
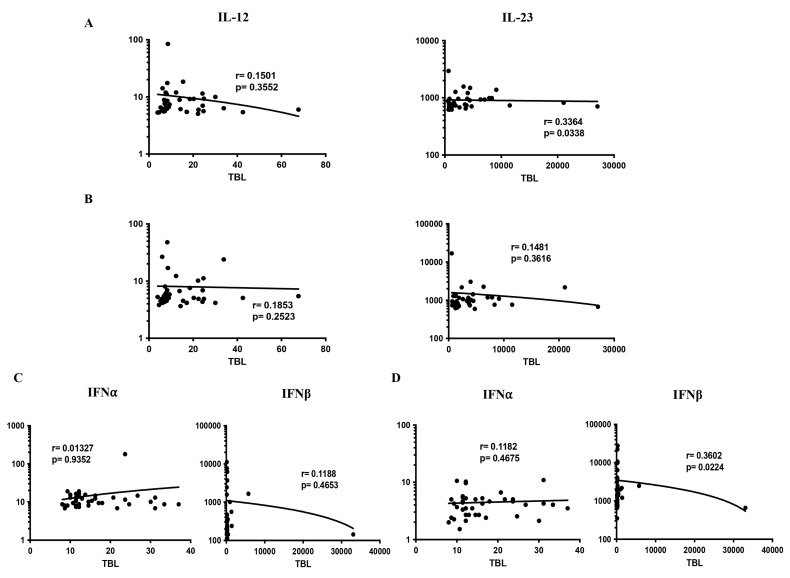
Relationship (correlation) of pro-inflammatory cytokines among the study groups. We determined the correlation ability of different (**A**–**D**) pro-inflammatory cytokines in TBL (*n* = 40) versus LTBI (*n* = 40) and TBL versus HC (*n* = 43) individuals. Each circle indicates a single individual, and the bar represents the geometric mean. *p*-values were analyzed using the Spearman rank correlation.

We measured the relationship of pro-inflammatory (IL-12, IL-23, IFNα, IFNβ) cytokines among TBL, LTBI, and HC individuals (Figure 4A–D). As shown in Figure 4, among the pro-inflammatory cytokines, IL-23 (r = 0.3364; *p* = 0.0338) had a significant positive correlation in TBL compared to the LTBI group, whereas IFNβ (r = 0.3602; *p* = 0.0224) had a positive correlation in the TBL compared to the HC groups. In contrast, the other pro-inflammatory cytokines, IL-12 (TBL versus LTBI and HC) and IL-23 (TBL versus HC), IFNα, IFNβ (TBL versus LTBI), and IFNα (TBL versus HC) did not exhibit a significant correlation between the study groups (Figure 4A–D). Thus, IL-23 and IFNβ pro-inflammatory cytokines significantly correlate with TBL disease.

## 4. Discussion

The correlates of protective immunity in TBL are still not well characterized, especially in the context of pro-inflammatory cytokines since their presence or absence exerts a balance between protective and destructive immune responses. It has been reported previously that mice lacking certain pro-inflammatory (IL-1α, IL-1β, IFNγ, TNFα, IL-17, and IL-12) cytokines were more susceptible to TB disease [7,11]. In TBL, some of the pro-inflammatory cytokines were significantly increased (IFNγ, TNFα, IL-17) and decreased (IL-1β) compared to PTB individuals [29]. Hence, we examined the systemic levels of other proinflammatory cytokines in our study (TBL, LTBI, HC) groups. Our results have shown that proinflammatory cytokines are altered (increased) and reversed significantly upon the completion of ATT in TBL disease.

IL-12 acts as an agonist for the immune responses and its deficiency results in the delayed function of lung DCs to prime naïve T cells [11]. IL-12 is also necessary for the protective IFNγ-mediated immune response against Mtb infection [17]. Finally, IL-12 is important in the differentiation and survival of memory CD4+ T cells and chemokine production [30,31]. Both mouse and human model data suggest that IL-12 is important in providing immunity to TB disease [32,33]. In humans, a lack of IL-12p40 cytokine exhibits an inherent susceptibility to Mtb infection. Similarly, the autosomal genes are linked with IL-12/IFNγ-related signaling and macrophage stimulation supported by IFNγ. After receiving BCG, people with mendelian susceptibility to mycobacterial disease (MSMD) demonstrate a vulnerability to Mtb infection and acquire BCG-osis due to IL-12Rβ1, IFNγ, and IL-12p40 deficiency [34]. In humans, toll-like receptors (TLRs) and other innate immune receptors allow NK cells to respond to free mycobacteria, thereby helping in the IFNγ release and cytolytic activity by IL-12 priming during the early DC period [35]. IL-12 mediates cellular activation by facilitating higher CCL1 and CCL17 chemokine production [31]. Similarly, STAT4 activation and IFNγ production was induced by IL-23 in the human T-cell population [36]. IL-23 also helps in the differentiation of Th17 cytokine-producing cells in humans [37,38] and augments the memory T cell responses [30]. IL-23 is important for the proper function of Th17 cells and IL-17 immunity against mycobacterial function [18]. Hence, the IL-12 family has a pivotal role in inflammatory responses and mycobacterial killing [39].

In our study, both cytokines were elevated in TBL individuals when compared to LTBI and HC groups, suggesting that they could potentially contribute to host defense against the TB infection or induce inflammation. Their significant alteration in the post-treatment time point depicts the direct association of these cytokines in TBL disease. Our lab previously reported different pro-inflammatory (TNFα, IFNγ, IL-1β, IL-6, IL-12, IL-17A, IL-17F) cytokines as markers of disease severity in PTB disease in comparison with LTBI and HC individuals [40].

We also studied the systemic levels of type 1 IFNs in TBL disease and compared them with LTBI and HCs because their function in the host response to TBL infection is poorly understood. A previous study reported the role of IFN-mediated transcriptional signature in circulating leukocytes of TB-affected patients, justifying the possible association of higher type 1 IFN signaling in active TB disease. Hence, interferon-activated gene signatures in both human and animal models have drawn significant attention in understanding the host response against Mtb infection due to their detrimental effects [23]. Human alveolar macrophages undergo premature cell death due to the presence of type 1 IFNs [24]. Apart from having a protective role in viral infection, they play a crucial function in the host response against bacterial infections [41,42]. Type I IFNs either promote or impair the pathogen control depending upon the infectious agent; specifically in the context of Mtb infection, it shows both protective and detrimental roles [23]. Similarly, IFNβ directly elevates the expression of anti-inflammatory cytokine levels and diminishes the pro-inflammatory cytokine levels [43]. The wild-type mice infected with an extremely virulent clinical strain of Mtb showed increased mRNA levels of IFNα compared to animals exposed to less active strains [44]. A previous study has shown that pre-treatment with IFNα increases IL-10 production and impairs bacteriostatic activity [45,46]. Diverse mechanisms have been provided to explain the importance of type I IFNs in exacerbating Mtb infection [9,42].

Our study also revealed that the TBL group has elevated IFNα and IFNβ cytokine levels compared to LTBI and HC individuals. This result indicates that type 1 IFNs might stimulate immunopathology in the context of TBL disease. To support the data, it was shown that Mtb infection significantly heightens the expression of type I IFN inducible genes in vitro [47,48] and diminishes the Th1-mediated immunity by decreasing IFNγ production and propagation by antigen-specific T cells [23,44,49]. As previously reported, higher levels of type I IFNs can promote TB disease pathogenesis via the direct activation of neutrophils [50]. Our data on TBL disease also indicates that increased levels of type 1 IFN might cause the disease pathogenesis in EPTB, which leads to uncontrolled Mtb growth at the affected lymph nodes.

Most importantly, it was reported that type I IFNs act as potential inhibitors of IL-1α and IL-1β cytokines, which are crucial for host protection against TB disease [24]. To support this fact, we showed previously that the TBL group has diminished systemic and antigen-presenting cytokines (IL-1β and IL-18) [51]. Similarly, another report also indicated that the inhibition of type 1 IFNs is associated with reduced IL-1β levels and higher 5-lipoxygenase expression [50]. Upon ATT, the circulating levels of IFNα levels were significantly decreased in the post-treatment group in comparison with the pre-treatment group. We also show that type 1 IFNs have a higher sensitivity and specificity in the TBL group discriminating against LTBI and HC individuals. We also report that both IL-12 and IFNβ were significantly correlated between the study groups. Our study has limitations in that the levels of pro-inflammatory cytokines have not been measured in the affected lymph nodes. In addition, apart from HIV, other confounding factors have not been screened in the study individuals. Moreover, the small sample size requires that these findings be validated in a study with larger cohorts.

## 5. Conclusions

This study describes specific pro-inflammatory cytokine patterns in TBL disease compared to LTBI and HC individuals, showing that specific cytokines are elevated in the systemic circulation. Some of these appear to be modulated by successful treatments, indicating a direct relationship with TB infection in LN. Hence, they can be used as a marker for disease pathogenesis and treatment monitoring, which might be useful in the development of targeted host-directed therapy.

## Figures and Tables

**Table 1:** Study population demographics.

Study Demographics	TBL	LTBI	HC
Number of subjects recruited (*n*)	40	40	43
Gender (M/F)	13/27	15/25	12/28
Median age in years (Range)	28 (18–51)	32 (21–62)	39 (23–55)
Lymph node culture grade (0/1+/2+/3+)	8/30/2/0	Not done	Not done
Interferon-gamma release assay	Not done	Positive	Negative

## Data Availability

All the data are available within the manuscript.
